# Extensive CGMD Simulations of Atactic PS Providing Pseudo Experimental Data to Calibrate Nonlinear Inelastic Continuum Mechanical Constitutive Laws

**DOI:** 10.3390/polym11111824

**Published:** 2019-11-06

**Authors:** Maximilian Ries, Gunnar Possart, Paul Steinmann, Sebastian Pfaller

**Affiliations:** Chair of Applied Mechanics, Friedrich-Alexander-Universität Erlangen-Nürnberg, Egerlandstrasse 5, 91058 Erlangen, Germany; gunnar.possart@fau.de (G.P.); paul.steinmann@fau.de (P.S.)

**Keywords:** molecular dynamics, simulation of polymers, mechanical properties of polymers, material characterization

## Abstract

In this contribution, we present a characterization methodology to obtain pseudo experimental deformation data from CG MD simulations of polymers as an inevitable prerequisite to choose and calibrate continuum mechanical constitutive laws. Without restriction of generality, we employ a well established CG model of atactic polystyrene as exemplary model system and simulate its mechanical behavior under various uniaxial tension and compression load cases. To demonstrate the applicability of the obtained data, we exemplarily calibrate a viscoelastic continuum mechanical constitutive law. We conclude our contribution by a thorough discussion of the findings obtained in the numerical pseudo experiments and give an outline of subsequent research activities. Thus, this work contributes to the field of multiscale simulation methods and adds a specific application to the body of knowledge of CG MD simulations.

## 1. Introduction and Outline

In contrast to continuum mechanics, particle-based simulation techniques provide insight into the processes taking place at the level of atoms or molecules. Thus, these approaches are well-suited to understand the behavior of material originating from the structures at very small length and time scales. However, when larger system sizes have to be employed, e.g., representative volume elements for composite materials with a representative number of inclusions, pure particle approaches may become computationally prohibitive due to the large number of degrees of freedom to be considered. In such problems, the combination of particle-based techniques with a continuum mechanical treatment has great potential to reduce the computational effort, but still allows for a sufficiently fine resolution in crucial regions of the domain of interest.

**Figure 1:**
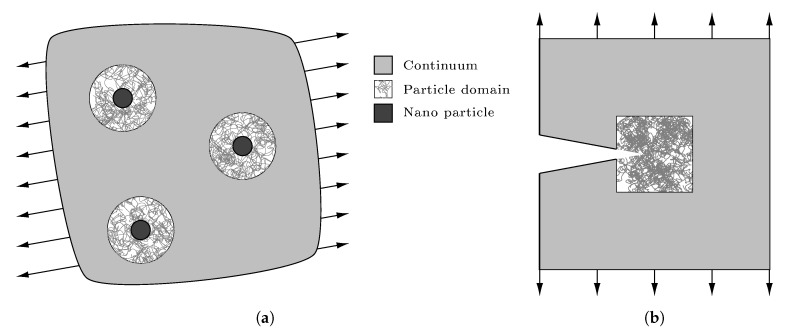
Examples for multiscale set-ups with fine resolution only in regions of interest: (**a**) polymer nanocomposite and (**b**) pre-cracked polymer sample.

Figure 1 sketches potential set-ups where only regions of specific interest are treated at the atomistic or molecular level: (a) displays a polymer nanocomposite with atomistic resolution only in the vicinity of the filler particles; (b) shows a pre-cracked sample with atomistic treatment only around the crack tip. Beyond these, a variety of applications is possible for the symbiotic usage of fine and coarse resolutions. Typically, the coarse scale (i.e., continuum mechanics) is applied in regions that are exposed to only moderate deformations, whereas the fine scale is required in parts of the domain where the material is subjected to large strains and stresses, which might arise from, e.g., discontinuities as sketched in Figure 1. To realize this kind of simulations, so-called partitioned-domain coupling approaches [1] are required which combine continuum mechanics and particle-based approaches. In the recent decades, a large number of multiscale simulation schemes has been proposed. Prominent examples are, e.g., presented and assessed in [2], but with focus on crystalline materials. Thus, they are less suited for the application in polymer systems. A comprehensive overview of multiscale methods focusing on polymer-matrix nanocomposites can be found in [3]. There, the authors conclude that the existing spectrum of molecular simulation methods still is not able to entirely describe the macroscopic behavior of polymer nanocomposites. To overcome this, they propose coupled atomistic-continuum simulation techniques as, e.g., the two-scale model by Semkiv et al. [4] to describe the viscoelastic behavior of elastomer nanocomposites. However, for concurrent multiscale modeling of amorphous polymers as sketched in Figure 1, partitioned-domain techniques as proposed in [5] have to employed. This kind of methods bases on the Arlequin approach introduced by Ben Dhia [6,7,8] and allows to model amorphous polymer systems concurrently. Inspired by this, Pfaller et al. [9] proposed the Capriccio method and proved it [10,11] to be a well-suited approach for polymers.

**Figure 2:**
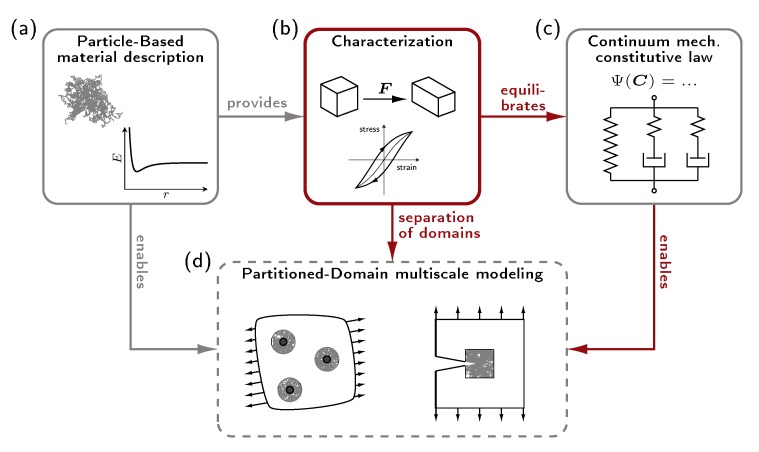
Schematic overview: Particle-based material description (**a**), characterization procedure (**b**), continuum mechanical constitutive law (**c**) and partitioned-domain multiscale investigations (**d**).

Such concurrent coupling schemes require matching material behavior at both scales of resolution, which, of course, has to take into account the specific limitations of the individual approaches. Typically, the continuum level should consider the material behavior only under moderate strains, and its constitutive relations have to be chosen accordingly. This requires a profound knowledge of the material behavior at the atomistic level, which, in the case of polymer systems, has no analytical link to a continuum mechanical description. Thus, a careful investigation of the material response under various loading conditions is required and forms the basis for the selection and specification of continuum mechanical constitutive laws. Furthermore, this knowledge is an important precondition for reliable separations into regions of fine and coarse resolution, which must be based on quantitative measures for the applicability of coarsening. In this contribution, we present a characterization methodology inspired from a continuum mechanical point of view [12] to investigate the material behavior at the atomistic level thoroughly. As sketched in Figure 2, this characterization procedure (b) plays an important, twofold role in concurrent multiscale modeling (d): Firstly, it provides the profound data base to derive continuum mechanical constitutive laws (c) from atomistic material descriptions (a) whenever, as described above, a direct analytical link between e.g., particle interactions and associated macroscopic quantities is not available. Secondly, the characterization procedure is crucial to separate the individual domains, i.e., to apply the coarse resolution only in parts with sufficiently low loads. In this context, also adaptive concepts to separate coarse and fine resolution rely on the findings obtained during the characterization. However, the results derived from the characterization procedure do not necessarily coincide with the outcome of mechanical testing. Thus, the continuum mechanical description desired here must not be regarded as a synonym for (i) large-scale continuum treatment or for (ii) experimentally determined material behavior, but serves as a partial substitution of the material description with less computational effort. If a link to experimental findings is aimed for, strategies as introduced by Li et al. [13] have to be employed. In their approach, macroscopic viscoelastic properties are predicted from structural findings obtained from atomistic simulations.

The characterization technique proposed in the present paper is inspired by the approach introduced by Rahimi et al. [14]. There, the authors analyze the mechanical properties of pure polystyrene (PS) and a nanocomposite consisting of PS and silica nanoparticles using Molecular Dynamics (MD). In order to increase the system size to be captured, they apply a coarse graining approach [15] allowing for the simulation of 300 polymer chains with 200 monomers each. They perform uniaxial tension tests to obtain Young’s modulus and Poisson’s ratio at different temperatures. From their results, however, it is obvious that the stress is not linearly related to the strain and, in addition, it is not clear whether the material behavior is purely elastic or contains also viscous and plastic contributions. Without restriction of generality, we adopt this coarse-grained molecular dynamics (CGMD) approach to develop the characterization methodology. However, its application to full atomistic descriptions is straightforward.

Based on this, our contribution is organized as follows: first, we describe the preparation of the MD systems to be investigated, followed by a detailed presentation of our simulation set up including MD as well as continuum mechanics aspects. In the main part, we propose a characterization methodology and thoroughly discuss the various deformation tests, such as time proportional and time periodic as well as relaxation and creep simulations, together with their results. The applicability of the MD-deformation data is shown by an exemplary the calibration procedure of a viscoelastic constitutive law of reduced complexity. Finally, we give conclusions and an outlook to further developments and possible applications.

## 2. Preparation of MD Systems

Since simulations of polymers at the atomistic level are computationally expensive and drastically limit the system sizes to be examined, we choose the coarse graining approach of Qian et al. [15] to ensure sufficiently large numbers of polymer chains and reasonable chain lengths at comparably small simulation times. The transfer of our methodology to fully atomistic systems is straightforward and thus justifies the choice of such a model system for method development. In the coarse graining approach employed here, each styrene monomer is substituted at its center of mass by a single superatom. In the case of atactic PS as considered here, two different types of superatoms are defined for the R and S enantiomers. As a result, the system size and thus the computational effort are reduced at least by a factor of 16 compared to the initial atomistic model.

The interactions between the superatoms have been derived for the coarse-grained system at a temperature of 590 K and a pressure of 101.3 kPa by Ghanbari et al. [16] via transformation of atomistic radial distribution functions into coarse-grained profiles with the help of the iterative Boltzmann inversion [17]. The resulting coarse-grained force fields describe the inter- and intramolecular nonbonded interactions as well as the pair interactions between two bonded superatoms and the interactions between three neighboring superatoms. However, the torsional potential is flat enough to be neglected in this apporach as shown by Milano and Müller-Plathe [18].

In general, coarse-grained potentials are only valid under the thermodynamic conditions under which they have been parameterized and cannot be transferred to e.g., other temperatures [14,19]. Additionally, as a consequence of the reduced system complexity and the softer interaction potentials, coarse-grained models always experience an implicit speed-up of their dynamics compared to their atomistic counterpart. In order to derive quantitative results of dynamic variables, the correct time mapping between coarse-grained and atomistic scale has to be found [20]. However, such a mapping is currently not available and not in the scope of our work. Thus, all subsequently derived quantities are evaluated on the coarse-grained scale, which accurately reflects our goal of reproducing the behavior of the particle-based model with a continuum description.

Despite the peculiarities of coarse-grained potentials, the model employed here has been proven to perform well in various applications, amongst those also in a concept to derive Poisson’s ratio locally [21], for a study of nanoparticles in a polymer melt [22], and for the investigation of polymer chain growth [23]. Beyond these, Rahimi et al. [14] investigated Young’s modulus and Poisson’s ratio, below glass transition temperature for this model and proposed a methodical reasonable correlation between Young’s moduli at atomistic and coarse-grained resolution by means of the associated glass transition temperatures.

In this contribution, we investigate pure atactic PS using the MD code IBIsCO developed by Karimi-Varzaneh et al. [24]. The investigated MD systems consist of 300 PS chains with 200 superatoms each, which are spatially distributed by a self-avoiding random walks algorithm implemented by Ghanbari et al. [16]. In all further steps, the number of particles is kept constant in the cubical simulation box that has an initial edge length of 30 nm.

Firstly, the molecules are given 5 ns to disentangle at constant volume and a temperature of 590 K (NVT ensemble) followed by an equilibration of 20 ns under constant temperature and atmospheric pressure $p\;=\;p_{\mathrm{atm}}$ (NPT ensemble). Secondly, the system is cooled down to 100 K at a constant cooling rate of 5 K $ns^{-1}$ and under constant atmospheric pressure. This is significantly below the glass transition temperature $T_{g}=170\;K$ in coarse-grained resolution [14], which relates to $T_{g}=370\;K$ obtained from atomistic simulations [25,26] and experiments [27]. Note that the applied cooling rate is extremely high and thus the obtained systems are assumed to behave rather like supercooled melt than real solid PS below $T_{g}$ [28]. In order to ensure sufficient equilibration, the system is kept at constant temperature and pressure for another 2 ns.

All the above steps are carried out under periodic boundary conditions with a time step of Δt=5fs. The pressure is kept constant at atmospheric level by a Berendsen barostat with pressure coupling time of 5 ps and isothermal compressibility of 1 $\times \;10^{-6}$ kPa, while the temperature is controlled by a Berendsen thermostat with temperature coupling time of 0.2 ps, as listed in Table 1.

In order to obtain statistically reliable results, a number of ten MD systems denoted as a measurement batch are prepared as described above and investigated in the following.

## 3. Model and Simulation Set Up

### 3.1. Continuum Mechanical Set Up

In this contribution, we want to consider uniaxial deformation, which is defined as an extension or compression of a body in one direction [29,30], while allowing free lateral contractions or expansions. We choose to prescribe the deformation and evaluate the corresponding stress response which is denoted as a strain controlled set up.

**Figure 3:**
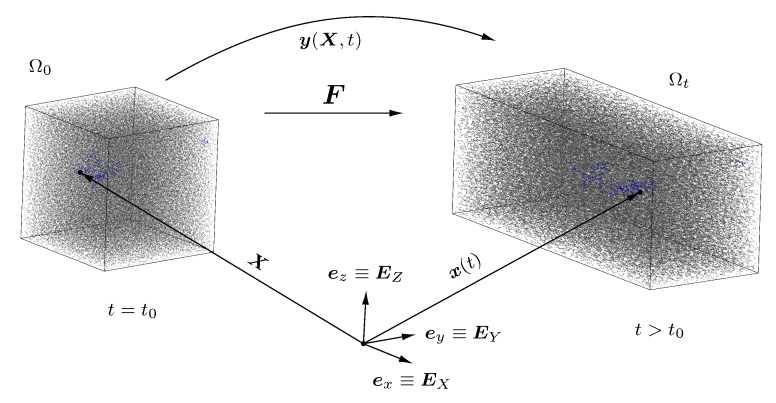
Continuum mechanical setting: Initial ($\Omega_{0}$) and current configuration ($\Omega_{t}$) of the MD simulation box, position vectors X and x(t), deformation map y(X,t) and deformation gradient F(X,t).

To embed this uniaxial deformation into the usual nonlinear continuum mechanics framework, we describe the physical body under consideration by means of the material configuration $\Omega_{0}$ (at initial time $t_{0}$) and the spatial configuration $\Omega_{t}$ the body occupies during the course of time for $t>t_{0}$, cf. Figure 3. Any point of the body is represented by its position vector X in the material configuration and the position vector x in the spatial configuration, which are defined in a Cartesian coordinate system with the basis vectors $E_{I}\equiv e_{i}$, i=x,y,z and I=X,Y,Z. Furthermore, we define the deformation map y(X,t) which maps the position vector X to its spatial counterpart x and introduce the deformation gradient $F\left(X,t\right)=\frac{\partial y(X,t)}{\partial X}$. The uniaxial deformation in $e_{i}$-direction then follows by means of the stretch $\lambda_{i}$ as $x_{i}=\lambda_{i}X_{i}$ (no summation).

### 3.2. Molecular Dynamics Set Up

**Figure 4:**
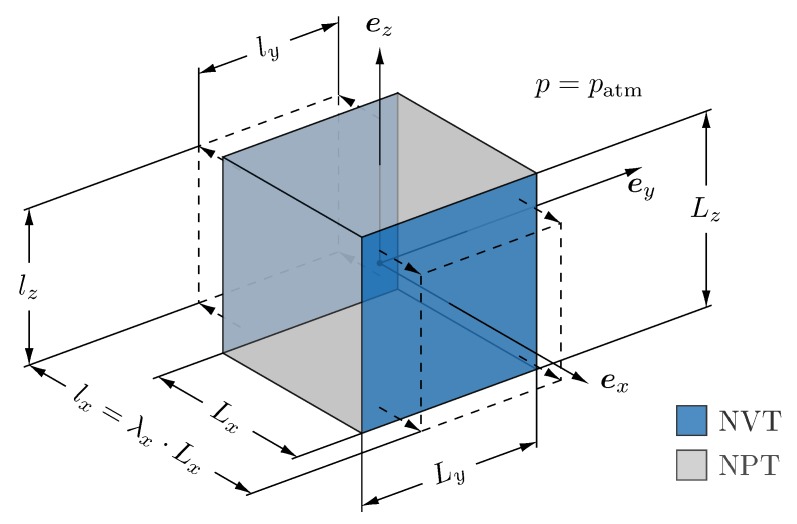
MD setup for uniaxial deformation in $e_{x}$ direction: Prescription of stretch $\lambda_{x}$ via NVT ensemble in tensile direction and application of atmospheric pressure $p_{\mathrm{atm}}$ by NPT ensemble in lateral directions.

To relate the continuum deformation to the deformation of an MD system, we have to establish a link between the continuum mechanics set up and the MD simulation box. To this end, we consider the undeformed continuum body to be a cube, which is oriented along the coordinate axes and deforms to a cuboid when subjected to uniaxial deformation, i.e., when a specific stretch $\lambda_{i}$ is prescribed. In this case, the associated MD simulation box (cf. Figure 4 with initial edge lengths $L_{x}=L_{y}=L_{z}$) has to be extended or compressed in one direction by prescribing the box length in loading direction, i.e., $\ell_{i}=\lambda_{i}L_{i}$ holds for the deformed edge length.

The application of strain-controlled uniaxial deformation in an isothermal MD simulation with a constant number of particles requires a specific ensemble set up as depicted in Figure 4. In tensile direction $e_{i}$, we prescribe the box length $\ell_{i}$ and thus mimic an NVT ensemble, whereas in the lateral directions we keep the pressure fixed at atmospheric level $p_{\mathrm{atm}}$ as it would be the case in an NPT ensemble.

The existing implementation of IBIsCO had to be extended to enable such uniaxial deformations.

The simulations are carried out under periodic boundary conditions and the relevant quantities, e.g., pressure and box dimensions, are sampled every 200 time steps. For the Berendsen thermostat and barostat, the parameters listed in Table 1 are used.

## 4. Uniaxial Deformation Simulations

**Figure 5:**
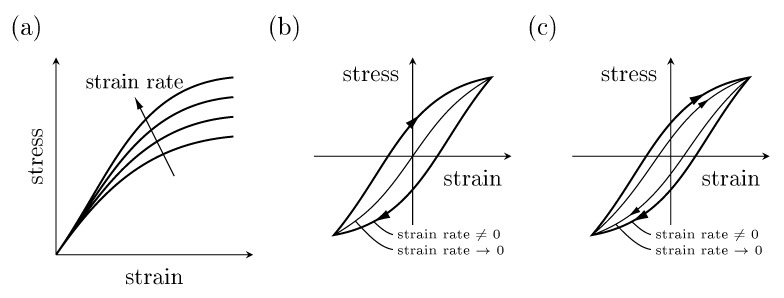
Schematic stress-strain relations: (**a**) strain rate dependency in time proportional tests, (**b**) cyclic behavior without quasi-static hysteresis and (**c**) cyclic behavior with quasi-static hysteresis.

A scheme to characterize materials based on experimental observations using the four theories of material behavior is introduced by Haupt [12]. The categorization is based on rate dependency and the existence of a so called equilibrium hysteresis for quasi-static loading [31] which will be denoted as quasi-static hysteresis in the following. These stress-strain relations are schematically shown in Figure 5. The categorization is depicted in Table 2 and identifies the material as elastic, plastic, viscoelastic or viscoplastic.

In order to determine these characteristics, time proportional and time periodic simulations are evaluated in the following. In addition, relaxation and creep tests are analyzed to corroborate the previous findings and to gain further insights about the behavior of PS.

### 4.1. Applied Deformation

In [14], Rahimi et al. investigated the mechanical properties of pure PS with MD systems similar to those used in our contribution. They conducted time proportional uniaxial tension tests independently along the three Cartesian axes on three different equilibrated MD systems. The maximum strain of 97% was applied with rates from 4.8 to 238%$ns^{-1}$. The results were derived by averaging over the three samples and three directions of deformation.

In contrast, we focus our investigations on much smaller strain rates of 0.1 to 20% $ns^{-1}$ which limits the examinable maximum strain in time proportional tests to 8%. The simulated time of 8 ns, however, is still significantly longer than the 2 ns of Rahimi et al. Furthermore, we examine ten slightly different MD systems and derive our final results with a more elaborate, step-wise averaging approach introduced below. Beyond that, we use time periodic uniaxial deformation, creep and relaxation tests to identify and investigate possible inelastic effects.

**Figure 6:**
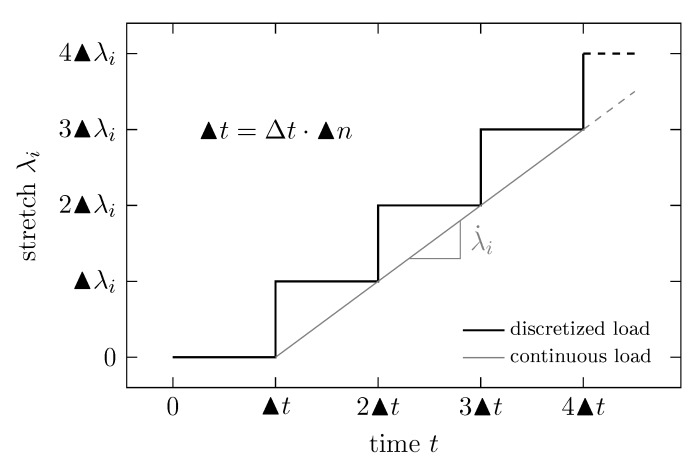
Discretization of continuos load: load step length ▴t, stretch increment $▴\lambda_{i}$, time step Δt and number of time steps per load step ▴n.

In the current implementation of IBIsCo, the deformation of the simulation box can be applied only step-wise. These load steps are characterized by their stretch increment $▴\lambda_{i}$ in tensile direction $e_{i}$ and their duration ▴t discretized in the number of time steps $▴n=▴t{\Delta t}^{-1}$ with time step Δt (cf. Figure 6).

In the time proportional uniaxial deformation tests, the stretch is applied with the constant rate in tensile direction $e_{i}$

(1) $$\begin{array}{c} {\dot{\lambda }}_{i}\left(t\right)=\frac{▴\lambda_{i}}{▴n\Delta t}=\mathrm{const}. \end{array}$$

**Figure 7:**
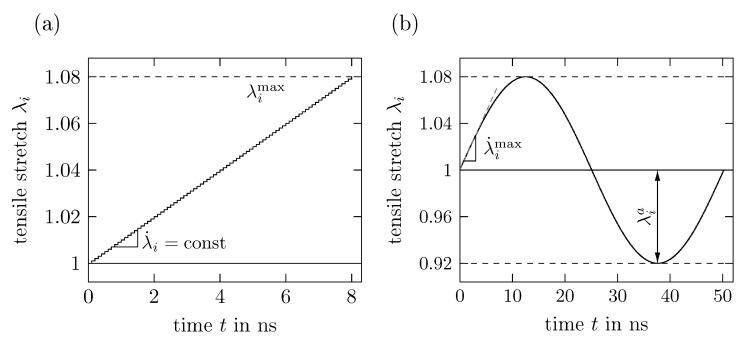
Applied stretch $\lambda_{i}$ in tensile direction $e_{i}$ over time *t* for (**a**) time proportional (i.e., noncyclic) loading with maximum stretch $\lambda_{i}^{\max}=1.08$ and stretch rate ${\dot{\lambda }}_{i}$ and (**b**) cyclic loading with stretch amplitude $\lambda_{i}^{a}=0.08$ and maximum stretch rate ${\left|{\dot{\lambda }}_{i}\right|}^{\max}=0.01ns^{-1}$.

The stretch increment and number of time steps are chosen as $▴\lambda_{i}=10^{-3}$ and ▴n= 40,000 time steps, respectively, and the stretch rate is set by adjusting Δt accordingly. As a consequence, the maximum stretch $\lambda_{i}^{\max}=1.08$ is reached after 80 load steps as depicted in Figure 7a. Analogously, uniaxial compression tests are derived by only inverting the direction of the stretch while leaving all other parameters fixed.

For the time periodic uniaxial deformation simulations, a sinusoidal function with a maximum stretch rate of ${{\dot{\lambda }}_{i}}^{\max}=0.01ns^{-1}$ is discretized with a time step of Δt=5fs into 20,000 time steps, so that $\left|▴\lambda_{i}\right|\leq 10^{-3}$ holds for the stretch increment. Each simulation consists of four full tension-compression cycles and stretch amplitudes $\lambda_{i}^{a}$ between 0.01 and 0.08 are investigated. The influence of the stretch rate is analyzed in subsequent tests with ${{\dot{\lambda }}_{i}}^{\max}=0.001ns^{-1}$ to 0.2 $ns^{-1}$ and a fixed amplitude of $\lambda_{i}^{a}=0.04$. The parameters used for time proportional and time periodic loading are summarized in Table 3.

The results will be discussed by means of the Green-Lagrange tensor E which, for the present load case, has the diagonal form

(2) $$\begin{array}{c} E=0.5\;\cdot \;\left[F^{t}\;\cdot \;F-1\right]=0.5\;\cdot \;\left[\lambda_{i}^{2}-1\right]e_{i}⊗e_{i} \end{array}$$

with deformation gradient F, stretch $\lambda_{i}$ and i=x,y,z.

### 4.2. Stress Evaluation

**Figure 8:**
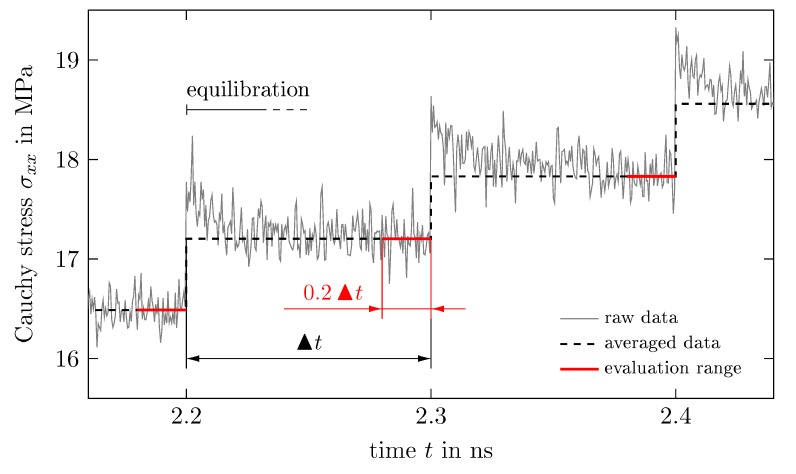
Stepwise filtering of stress in each load step: Average stress derived in evaluation range of 0.2▴t is projected as constant for the whole load step length ▴t.

The Cauchy stress tensor σ can be derived from the current pressure tensor p, which is sampled every 200 time steps by $\sigma =-\left[p-p_{\mathrm{atm}}\;1\right]$ with unit tensor 1. However, the pressure is subjected to very strong fluctuations and therefore has to be smoothed. This is realized according to Figure 8: in each load step, a specific stress is obtained by the mean over the evaluation range comprising the last 20% of data points. This procedure filters out the equilibration of the system which takes place in each load step and which is obvious from Figure 8. Furthermore, we thus reduce the number of data points significantly, since not the entire stress evolution in a load step has to be stored but only a data tuple comprising the load step strain and the associated mean stress.

All the resulting data discussed in the following represents an average over the measurement batch comprising ten MD systems (cf. Section 2) that is additionally smoothed using the stepwise filtering introduced above.

### 4.3. Time Discretization

**Figure 9:**
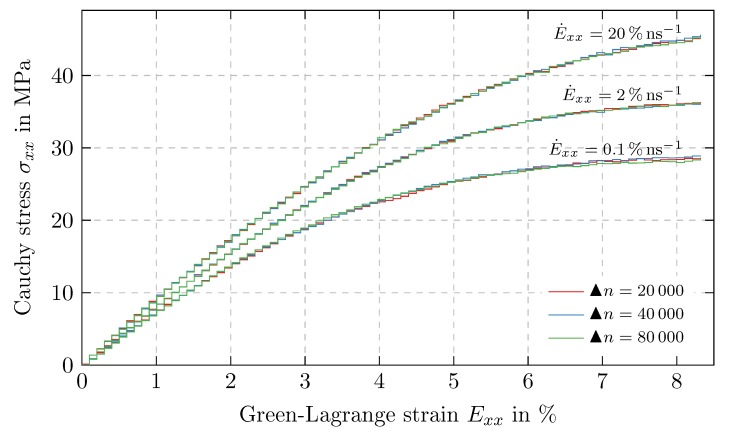
Influence of time discretization: Cauchy stress in tensile direction $\sigma_{xx}$ over Green-Lagrange strain $E_{xx}$ in tensile direction for time proportional (i.e., noncyclic) uniaxial tension tests with different time discretizations parametrized by the number of time steps per load step ▴n=20,000, 40,000 and 80,000 in combination with strain rates of 0.1% $ns^{-1}$, 2% $ns^{-1}$, 20% $ns^{-1}$.

To determine the influence of the time discretization, time proportional uniaxial tension tests are carried out in $e_{x}$-direction with ▴n= 20,000, 40,000 and 80,000 time steps. Three different ${\dot{E}}_{xx}$ are considered which are set by adjusting Δt according to (1), while $▴\lambda =10^{3}ns^{-1}$ remains unchanged. The resulting Cauchy stresses in tensile direction $\sigma_{xx}$ are shown in Figure 9, proving that the time discretization has no significant influence on the final results of the simulations.

## 5. Results

### 5.1. Isotropy

**Figure 10:**
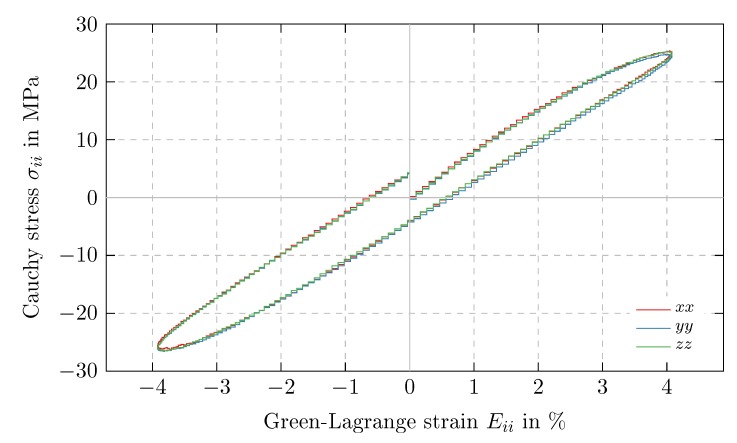
Dependency on load direction: Cauchy stress $\sigma_{ii}$ over Green-Lagrange strain $E_{ii}$ for time periodic (i.e., cyclic) uniaxial tension tests in tensile direction $e_{i}$ with i=x,y,z, strain amplitude $E_{ii}^{a}=4\%$ and strain rate ${\dot{E}}_{ii}=1\%\;ns^{-1}$.

In order to investigate the directional dependency of the mechanical behavior of PS, time periodic uniaxial deformation tests are performed independently in $e_{x}$-, $e_{y}$- and $e_{z}$-direction with a maximum strain amplitude of $E_{i}^{a}=4\%$. Since there is no significant deviation between the resulting stress-strain curves (cf. Figure 10), the material can be considered isotropic. As a consequence, all further investigations are merely conducted in $e_{x}$-direction.

**Figure 11:**
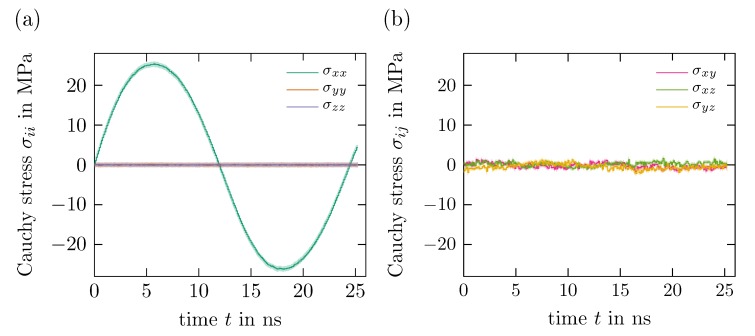
Comparison of the components of the Cauchy stress tensor σ over time *t* for a cyclic uniaxial tension test in tensile direction $e_{x}$, strain amplitude $E_{xx}^{a}=4\%$ and strain rate ${\dot{E}}_{xx}=1\%\;ns^{-1}$: (**a**) normal stress components $\sigma_{xx}$, $\sigma_{yy}$, $\sigma_{zz}$ and (**b**) shear stress components $\sigma_{xy}$, $\sigma_{xz}$, $\sigma_{yz}$ with respective standard deviation.

In comparison to the stress component in tensile direction, the other entries of σ are significantly smaller, as expected from an uniaxial tension test. This is exemplary shown in Figure 11 for the cyclic deformation in $e_{x}$-direction discussed above. While the tensile stress follows the sinusoidal course of the applied strain, the stress components $\sigma_{yy}$ and $\sigma_{zz}$ in lateral direction are approximately zero, ±0.15MPa. There are, however, fluctuations of the shear stress components (cf. Figure 11b) of ±2MPa which could be explained by natural fluctuations intrinsic to dynamic systems. Since these deviations are comparatively small, they will be neglected and thus only the stress component in tensile direction is considered in the following.

### 5.2. Results of Time Proportional Tests

**Figure 12:**
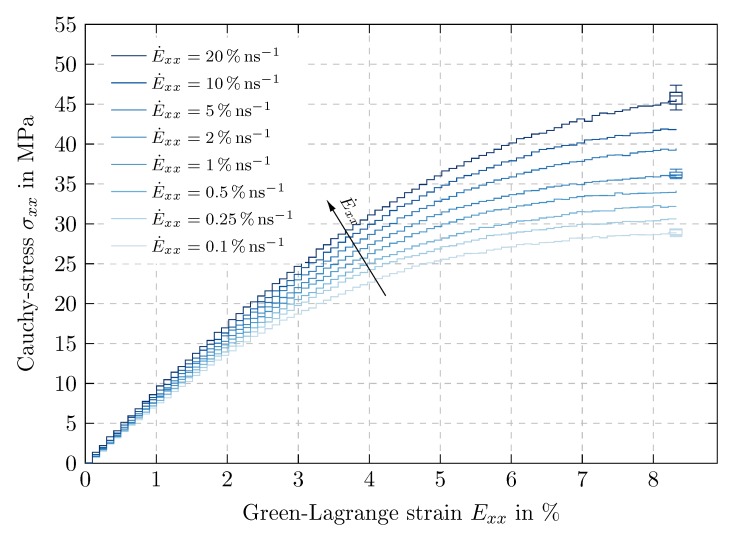
Influence of strain rate: Cauchy stress in tensile direction $\sigma_{xx}$ over Green-Lagrange strain in tensile direction $E_{xx}$ for time proportional uniaxial tension tests with maximum strain $E_{xx}^{\max}=8.036\%$ and initial strain rates from ${\dot{E}}_{xx}=0.1\%\;ns^{-1}$ to 20% $ns^{-1}$, distribution over measurement batch visualized by box plots.

The stress-strain curves of time proportional uniaxial tension tests in $e_{x}$-direction with different strain rates are compared in Figure 12. Each of them shows a linear course up to 1% strain and subsequently flattens noticeably. However, a distinguished yield point cannot be identified.

The material behaves stiffer at higher strain rates and thus indicates a clear dependence on the load rate. When subjected to a fast deformation, the polymer has only little time to recover its molecule chains which results in increased stresses [32].

The distribution of the simulation results over the measurement batch is visualized by box plots [33]. Since this dispersion is considerably small, especially in comparison with the influence of the strain rate, the results are corroborated statistically.

**Figure 13:**
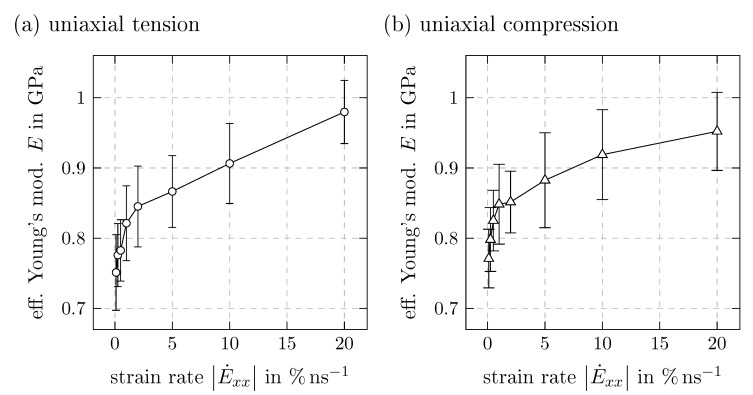
Effective Young’s modulus *E* derived as the slope of the secant between 0 and 1% strain from time proportional uniaxial tension (**a**) and compression tests (**b**) with strain rates from $\left|{\dot{E}}_{xx}\right|\;=\;0.1\%\;ns^{-1}$ to 20% $ns^{-1}$ with respective standard deviation.

The initial slope *E* of the secant between 0 and 1% strain from uniaxial tension and compression tests can be considered as an effective Young’s modulus and is plotted in Figure 13. In both load cases, this quantity shows a strong increase up to a strain rate of 1% $ns^{-1}$ and then flattens progressively. Due to the coarse graining, the obtained *E* is much lower than values from atomistic simulations [26] or experiments [34,35].

**Figure 14:**
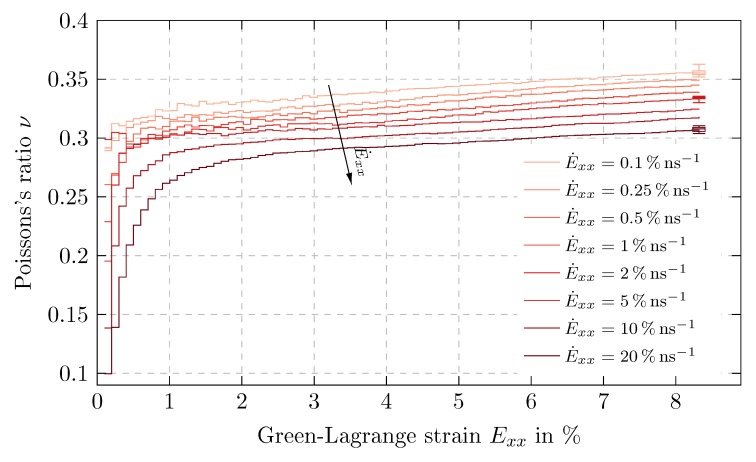
Influence of strain rate: Poisson’s ratio ν over Green-Lagrange strain $E_{xx}$ in tensile direction for time proportional uniaxial tension tests with maximum strain $E_{xx}^{\max}=8.036\%$ and initial strain rates from ${\dot{E}}_{xx}=0.1\%\;ns^{-1}$ to 20% $ns^{-1}$, distribution over measurement batch visualized by box plots.

The Poisson’s ratio ν is determined only at the end of each load step to exclude the respective stepwise equilibration of the systems. Figure 14 compares ν as a function of $E_{xx}$ for different strain rates. In all simulations, higher extension rates lead to a lower lateral contraction and hence a smaller Poisson’s ratio. The initial values of ν range from 0.1 to 0.29 for strain rates of 20% $ns^{-1}$ to 0.1% $ns^{-1}$, respectively. At higher deformations rates, we assume that the polymer chains do not have sufficient time to sample conformational space and remain in an unrelaxed state. Overall, this results in a more pronounced lack of lateral strain for higher load rates and thus a smaller ν.

Subsequently, all ν-curves increase exponentially up to 3% strain while the deviation between the different strain rates reduces. After that, the Poisson’s ratio increases linearly with the same slope of $3.3\;\cdot \;10^{-3}$ ${\%}^{-1}$ for all strain rates resulting in ν=0.31 and 0.37 for 20% $ns^{-1}$ to 0.1% $ns^{-1}$, respectively.

Additionally, box plots again show small variability of the data and thus confirm the statistical reliability of the results.

The derived characteristics of *E* and ν are in good agreement with the findings of Rahimi et al. [14].

### 5.3. Results of Time Periodic Tests

**Figure 15:**
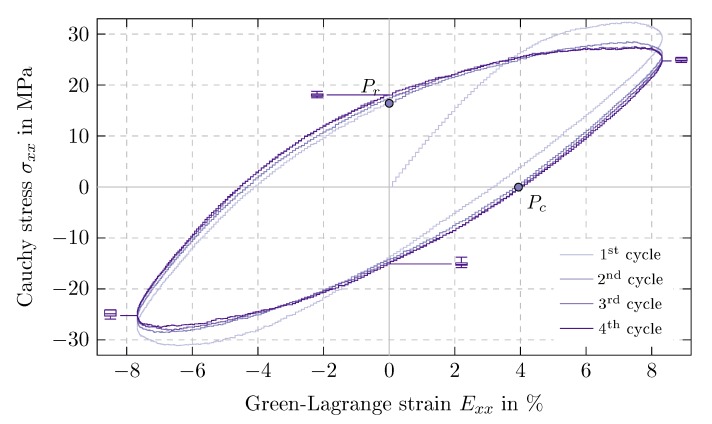
Time periodic uniaxial tension: Cauchy stress $\sigma_{xx}$ over Green-Lagrange strain $E_{xx}$ in tensile direction for strain amplitude $E_{xx}^{a}=8\%$ and starting points $P_{r}$ and $P_{c}$ for stress relaxation test and creep simulations, respectively, distribution over measurement batch for 4th cycle visualized by box plots.

The resulting stress-strain hystereses of the time periodic uniaxial tensile tests are displayed in Figure 15, exemplary for $E_{xx}^{a}=8\%$. In the first cycle, the stress extrema of 32.3 MPa and −31.1 MPa are obtained. Subsequently cyclic softening [36] occurs, resulting in a stabilized hysteresis for the third cycle. This behavior is also observed qualitatively for the other strain amplitudes investigated here. From now on, only the stabilized hysteresis, i.e., the 3rd and 4th cycle, are considered.

Note that the end of the first hysteresis and the subsequent zero crossing of the stress, denoted by $P_{r}$ and $P_{c}$, are the starting points for the relaxation and creep tests in Section 5.4, respectively.

In the following, the influences of the applied loading on the maximum stress $\sigma_{xx}^{\max}$, the dissipated energy density $d_{0}^{\mathrm{cyc}}$ and the loss tangent tanϕ are investigated. The time periodic loading is mainly characterized by the strain amplitude $E_{xx}^{a}$ and the maximum strain rate ${\dot{E}}_{xx}^{\max}$. In order to identify possible relations, the dependencies on strain amplitude and strain rate have to be isolated. Thus simulations with different amplitudes but at the same strain rate ${\dot{E}}_{xx}^{\max}=1\%\;ns^{-1}$ and data obtained at different rates but with the same amplitude $E_{xx}^{a}=4\%$ are evaluated. Additionally, the following plots feature basic functions fitted to the displayed data to highlight the type of relation between the discussed quantities. This, however, does not prove a direct dependency between them.

**Figure 16:**
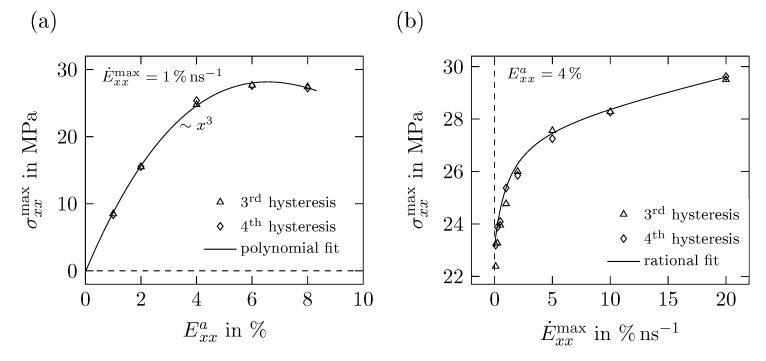
Maximum Cauchy-stress $\sigma_{xx}^{\max}$ obtained in cyclic uniaxial tension tests for (**a**) different strains amplitudes $E_{xx}^{a}$ with ${\dot{E}}_{xx}^{\max}=1\%\;ns^{-1}$ and (**b**) different maximum strain rates ${\dot{E}}_{xx}^{\max}$ with $E_{xx}^{a}=4\%$.

Figure 16a depicts the relation between $E_{xx}^{a}$ and the maximum stress $\sigma_{xx}^{\max}$, which increases up to a maximum of 27.6 MPa at 6% strain matching a cubic parabola. Although the stress obtained at 8% strain is slightly lower, it is possible that a threshold is reached around 27.5 MPa and $\sigma_{xx}^{\max}$ would not increase further if larger strains were applied.

The influence of ${\dot{E}}_{xx}^{\max}$ on the maximum stress $\sigma_{xx}^{\max}$ is depicted in Figure 16b. In general, larger $\sigma_{xx}^{\max}$ are obtained for increasing ${\dot{E}}_{xx}^{\max}$. However, this influence can be divided into two parts: firstly, for rates of 0.1% to 2% a steep rise of $\sigma_{xx}^{\max}$ is obtained which is then shifting towards a linear increase with significantly smaller gradient for strains rates between 5% and 20%.

An important measure for the inelasticity of a material is the dissipated energy density $d_{0}^{\mathrm{cyc}}$ which is represented by the area comprised by stress-strain-hystereses. The respective strain and stress measures, however, cannot be chosen arbitrarily but have to be work conjugated, i.e., $\left(P,\dot{F}\right)$, (σ,d) and $\left(S,\dot{E}\right)$ [37].

Using the last pair of Piola-Kirchhoff stress tensor S and Green-Lagrange strain E, the internal power density per unit volume in the undeformed configuration can be written as

(3) $$\begin{array}{c} p_{0}^{\mathrm{int}}=S:\dot{E} \end{array}$$

according to [29,38]. With a time integration of $p_{0}^{\mathrm{int}}$ over one deformation cycle (denoted by $\int_{\mathrm{cyc}}$) the part accounting for the stored energy density vanishes and hence

(4) $$\begin{array}{c} \int_{\mathrm{cyc}}p_{0}^{\mathrm{int}}\;dt=\int_{\mathrm{cyc}}S:\dot{E}\;dt=\int_{\mathrm{cyc}}S:dE=:d_{0}^{\mathrm{cyc}} \end{array}$$

yields the dissipated energy density $d_{0}^{\mathrm{cyc}}$ per cycle.

In the present case, E is already known and $S=JF^{-1}\;\cdot \;\sigma \;\cdot \;F^{-t}$. This simplifies to $S_{xx}=\lambda_{x}^{-1}\lambda_{y}\lambda_{z}\sigma_{xx}$ since $\sigma_{xx}$ is the only non-zero component of the Cauchy stress tensor as shown in Figure 11.

**Figure 17:**
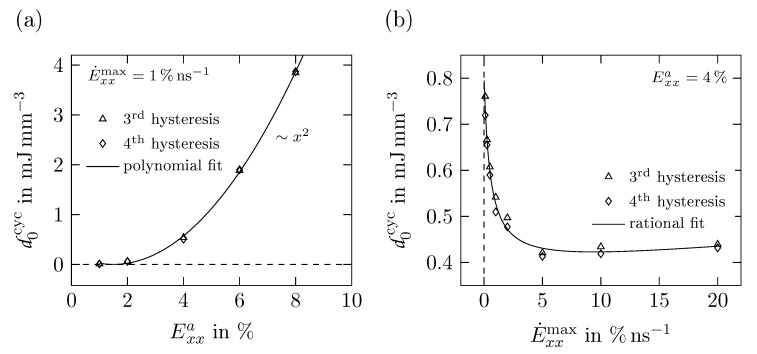
Dissipated energy density $d_{0}^{\mathrm{cyc}}$ derived from cyclic uniaxial tension tests for (**a**) different strains amplitudes $E_{xx}^{a}$ with ${\dot{E}}_{xx}^{\max}=1\%\;ns^{-1}$ and (**b**) different maximum strain rates ${\dot{E}}_{xx}^{\max}$ with $E_{xx}^{a}=4\%$.

The dissipated energy densities $d_{0}^{\mathrm{cyc}}$ obtained from simulations with different strain amplitudes at ${\dot{E}}_{xx}^{\max}=1\%\;ns^{-1}$ are presented in Figure 17a. For small amplitudes up to 2%, very little energy is dissipated indicating an almost purely elastic behavior in this range. The fitted curve reveals a quadratic rise of $d_{0}^{\mathrm{cyc}}$ for larger $E_{xx}^{\max}$, which corresponds to an increase in the inelasticity of the material.

In contrast to that, Figure 17b displays the influence of the maximum strain rate ${\dot{E}}_{xx}^{\max}$ on $d_{0}^{\mathrm{cyc}}$. The largest amount of energy is dissipated for strain rates approaching an asymptote of 0% $ns^{-1}$ proving the existence of quasi-static hystereses. However, $d_{0}^{\mathrm{cyc}}$ drops rapidly for increasing ${\dot{E}}_{xx}^{\max}$ until reaching a minimum at around $10\%\;ns^{-1}$. Additionally, a slight increase of $d_{0}^{\mathrm{cyc}}$ is observed for larger strain rates. As mentioned above, the slower the strain is applied, the more time the polymer has to rearrange its chains and thus to dissipate more energy.

Another significant quantity is the loss tangent tanϕ which measures the ratio of dissipated to stored energy for viscoelastic materials [39]. In this context, ϕ denotes the phase offset between stress and strain with ϕ=0 representing purely elastic behavior, whereas a phase shift of $\pm \frac{\pi }{2}$ characterizes purely viscous material behavior. The stress measures σ(t), P(t) and S(t) are all in phase and thus the choice of stress measure is indifferent. The phase offset is derived by fitting $\sigma_{xx}\left(t\right)$ with a sinusoidal function, comparing the resulting function with the applied strain $E_{xx}\left(t\right)$ and a subsequent averaging over all ten investigated MD configurations.

**Figure 18:**
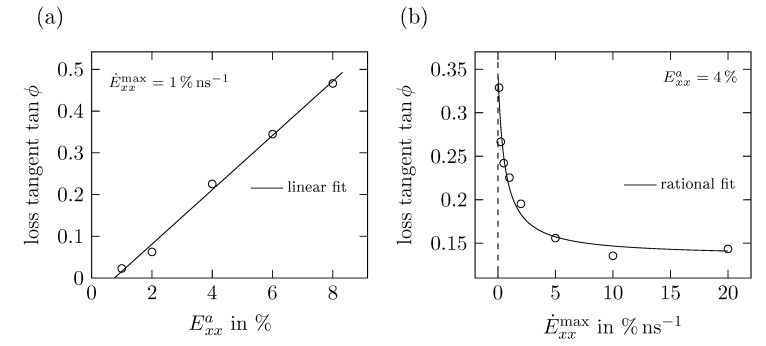
Loss tangent tanϕ with phase offset ϕ derived from cyclic uniaxial tension tests for (**a**) different strains amplitudes $E_{xx}^{a}$ with ${\dot{E}}_{xx}^{\max}=1\%\;ns^{-1}$ and (**b**) different maximum strain rates ${\dot{E}}_{xx}^{\max}$ with $E_{xx}^{a}=4\%$.

As shown in Figure 18a, the loss tangent tanϕ increases in a wide range linearly with increasing strain amplitude. However, for tensile strains lower than 1% we expect tanϕ to be zero or close to zero.

Figure 18b shows the influence of ${\dot{E}}_{xx}^{\max}$ on tanϕ, which is very similar to the relation between ${\dot{E}}_{xx}^{\max}$ and $d_{0}^{\mathrm{cyc}}$, cf. Figure 17. For small ${\dot{E}}_{xx}^{\max}$, the loss tangent approaches values up to 0.33, which coincides with a phase shift of approximately 18${}^{\circ }$ between stress and strain. On the other hand, the loss tangent seems to converge for large deformation rates to a constant value of 0.14 representing a phase shift of 8${}^{\circ }$.

### 5.4. Stress Relaxation and Creep Tests

**Figure 19:**
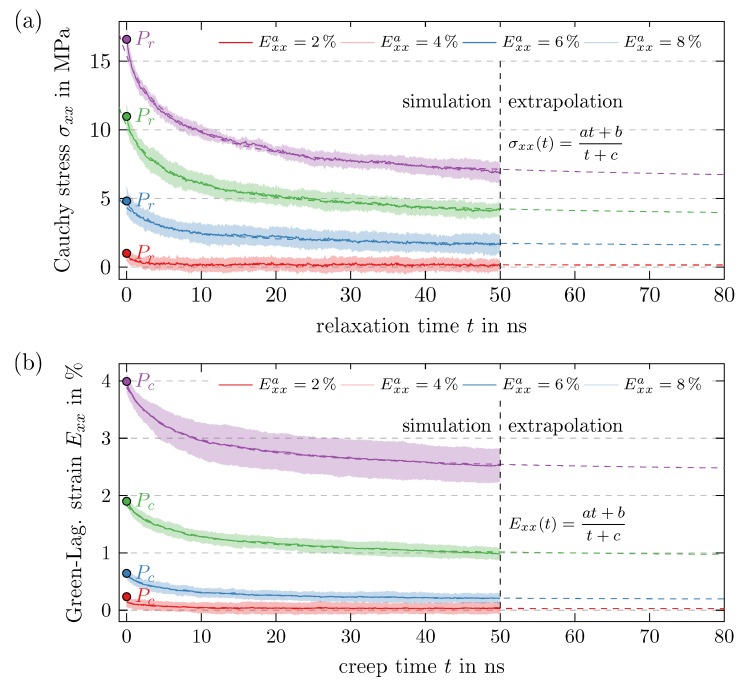
Identification of plasticity on the basis of (**a**) relaxation tests with $E_{xx}=0\%=const$ and (**b**) creep tests with $\sigma_{xx}=0M\mathrm{Pa}=const$, both with preceding cyclic loading with different strain amplitudes $E_{xx}^{a}$, extrapolation of simulation data with rational functions, distribution over measurement batch represented by standard deviation.

So far, it has been shown that the material behaves elastic for small strains and has additional inelastic components which can be explained either by viscous or plastic effects [36]. In the following, this will be examined more detailed through relaxation and creep tests. Since these load cases are not discretized in load steps, the step-wise filtering introduced in Figure 8 cannot be used. Instead, a simple Savitzky-Golay filter [40] with cubic polynomials and a frame length of 251 data points is applied for the evaluation of the resulting stress and strain. In order to assess the material response for t→∞, the obtained results are fitted and extrapolated (cf. Figure 19) with rational functions $f\left(t\right)=\frac{at+b}{t+c}$ with parameters a, *b* and *c* listed in Table 4. Note that a=f(t→∞) and $bc^{-1}=f\left(t=0\right)$.

For the relaxation tests, a full sinusoidal tension-compression cycle with strain amplitude $E_{xx}^{a}$ is carried out before the system is held for 50 ns at a constant strain of 0%. The starting point $P_{r}$ (cf. Figure 15) is chosen, to eliminate the stresses due to elasticity. Thus, the remaining stress comprises only plastic and viscous parts and the latter is expected to vanish completely after a sufficiently long relaxation time. However, Figure 19a shows that the resulting stresses vanish only for small preceding deformations and thus plastic effects occur for larger $E_{xx}^{a}$.

The creep tests, on the other hand, consist of a full sinusoidal tension-compression cycle that is continued until $\sigma_{xx}=0\;M\mathrm{Pa}$ is reached at $P_{c}$ (cf. Figure 15). Thus, there cannot be an elastic strain and the deformation has to be composed only of viscous and plastic parts. Subsequently, the system is held at this stress level for 50 ns and the viscous part of the deformation is expected to vanish. Again, this is only observed for small preceding strain amplitudes (cf. Figure 19b). As a consequence, the remaining strain for larger $E_{xx}^{a}$ can only be explained by plastic effects.

## 6. Exemplary Calibration of Continuum Mechanical Constitutive Laws by Means of MD-Data

To give an outlook on the subsequent steps of this work and to demonstrate the applicability of the MD-deformation data presented above for the calibration of continuum mechanical constitutive laws, an instructive example is discussed in the following. To this end, geometry and deformation history of the MD-simulation are precisely reproduced by a finite element model. The material parameters of the FE-model are then iteratively adjusted until its force response matches that of the MD-simulation as good as possible. Such procedures are known as FE-based inverse parameter identification and are available in a multitude of different formulations. The FE-model used here has been realized with the commercial software Abaqus, the optimizer comes with Matlab and is based on a gradient descent method. Although the material model used for the FE-simulation is a geometrically nonlinear viscoelastic formulation it turns out to be not yet complex enough to capture all types of inelasticity contained in the MD-data, but still allows for a semi-quantitatively reproduction of the force-displacement behavior. More complex material models and a comprehensive inverse parameter identification procedure will be presented in a follow-up contribution.

**Figure 20:**
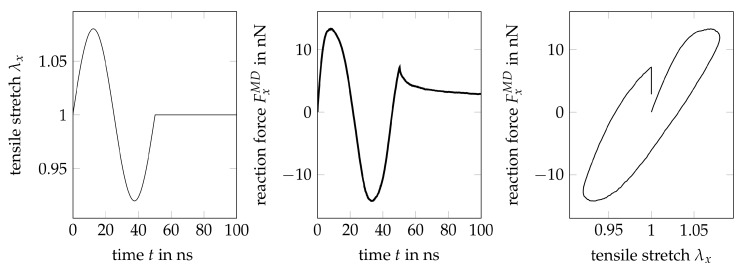
Uniaxial stretch history used in MD-simulation and reproduced by the FE-model (**left**), force history resulting from MD-simulation (**center**), building the goal function for the parameter optimization, corresponding force-stretch curve (**right**).

The governing parameters (stiffnesses, relaxation times…) of the material model are optimized with respect to the force-time curve obtained from a uniaxial cyclic MD simulation as shown in Figure 7b, i.e., we consider a single sinusoidal loading-unloading cycle with stretch amplitude $\Delta \lambda_{x}=\pm 0.08$ and maximum stretch rate $|{\dot{\lambda }}_{x}^{\max}|=0.01\;{ns}^{-1}$. To incorporate some relaxation information into the goal function of the optimization, the loading-unloading cycle is extendend by a holding phase of 50 ns after the compressive stretch has been reduced to zero, i.e., up from point $P_{r}$, cf. Figure 15. Stretch history and corresponding force response as resulting from the MD-simulation are depicted in Figure 20.

The above data indicate a nonlinear inelastic material behavior. One can observe nonlinear slopes during loading and unloading, which, together with strains larger than 3%, justifies the application of a hyperelastic material model and a geometrically nonlinear formulation. Furthermore, relevant stress relaxation is included, i.e., the material model has to include viscous mechanisms like dampers. Finally, the relaxation of the reaction force does not converge to zero, which implies plasticity and the necessity to further extend the material model complexity accordingly. From the point of view of a mechanical engineer it is remarkable that the material behavior obtained by MD is highly inelastic and complex, although all particle interactions are modelled exclusively by potentials, i.e., elastically.

### 6.1. A Hyper-Viscoelastic Material Law: Neo-Hooke with Linear Maxwell Element(s)

**Figure 21:**
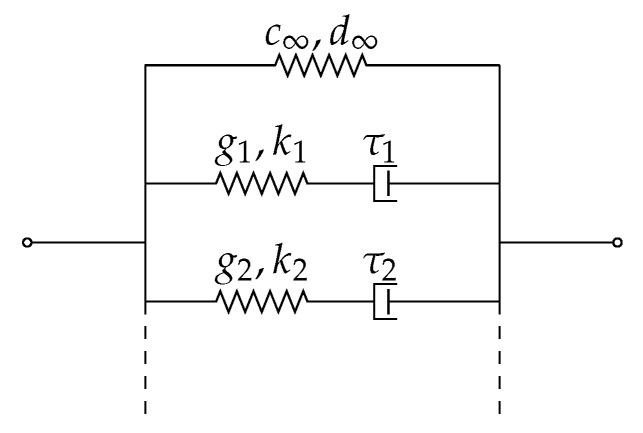
Rheological representation of the viscoelastic material model. $c_{\infty }$ and $d_{\infty }$ denote deviatoric and volumetric equilibrium moduli, respectively, i.e., at strain rate zero. $\tau_{i}>0$ are the relaxation times with which the fractions $g_{i}$ and $k_{i}\in \left[0,1\right)$ of total deviatoric and total volumetric stresses are dissipated. The deviatoric modulus of the spring in Maxwell element *i*, for example, then follows from $c_{i}=g_{i}\left[c_{\infty }+\sum_{j\neq i}c_{j}\right]/\left[1-g_{i}\right]$.

In order to not exceed the scope of this article, a material law of reduced complexity will be used in the following to demonstrate the calibration procedure. Plastic deformations are thereby neglected for simplicity, i.e., the model is limited to hyperelasticity, but allows for viscous effects. With the help of simple rheological models, this can be represented as a parallel connection of a nonlinear spring with a suitably chosen number of Maxwell elements, cf. Figure 21, which is also known as Prony series expansion. While the springs of the Maxwell elements also behave nonlinearly, the dampers are kept linear for simplicity, i.e., their viscosities are independent of the strain rate. Using several Maxwell elements with different relaxation times then still allows to approximate nonlinear viscous behavior.

The springs, i.e., the elastic parts of the material response, are described by the so-called Neo-Hooke model, which is a simple, yet widely used model for polymeric materials. Among other advantages, it has the charm to follow from statistical considerations on the micromechanical behavior of three-dimensional networks of Gaussian random walk chains, cf. e.g., [41]. The strain energy density function of the Neo-Hooke model is given by

(5) $$\Psi =c\left[{\bar{I}}_{1}-3\right]+\frac{1}{d}{\left[J-1\right]}^{2}$$

and depends on volumetric deformations via J=detF, i.e., the determinant of material gradient F=∂φ/∂X of the nonlinear deformation map φ. The strain energy density Ψ furthermore depends on isochoric deformations via ${\bar{I}}_{1}$, the first invariant and trace of the isochoric right Cauchy-Green tensor $\bar{C}={\bar{F}}^{T}\bar{F}$, whereby $\bar{F}=\left[J^{-1/3}I\right]F$. This first invariant can also be expressed as ${\bar{I}}_{1}={\bar{\lambda }}_{1}^{2}+{\bar{\lambda }}_{2}^{2}+{\bar{\lambda }}_{3}^{2}$ in terms of the isochoric principal stretches ${\bar{\lambda }}_{i}=J^{-1/3}\lambda_{i}$. Stress tensor and tangent operator required for a finite element implementation of this material model then follow as the derivatives with respect to the strain tensor, the details can be found e.g., in [41] and are omitted here.

The governing material parameters *c* and *d* are related to commonly used elasticity constants via:

(6) $$\begin{array}{cc} \mathrm{shear}\;\mathrm{modulus}:\;\mu & =2c \end{array}$$

(7) $$\begin{array}{cc} \mathrm{bulk}\;\mathrm{modulus}:\;k & =\frac{2}{d} \end{array}$$

(8) $$\begin{array}{cc} \mathrm{Young}’s\;\mathrm{modulus}:\;E & =\frac{36c}{6+2dc}\; \end{array}$$

(9) $$\begin{array}{cc} \mathrm{Poisson}’s\;\mathrm{ratio}:\;\nu & =\frac{6-4cd}{6+4cd} \end{array}$$

For the sake of convenience, all optimizations discussed in the following have been carried out in terms of Young’s modulus *E* and with a fixed Poisson’s ratio ν=0.325. A simultaneous optimization of ν is possible if lateral contraction data as available from the MD-simulations would be incorporated into the goal function. Similar to the treatment of plasticity, this is postponed to a follow-up contribution.

To extend the Neo-Hooke model by viscous behavior in the sense of Figure 21, the material parameters are now considered as time-dependent functions given by the following definitions

(10) $$c\left(t\right)=c_{0}\left[1-\sum_{i=1}^{n}g_{i}\left[1-\exp\left(-\frac{t}{\tau_{i}}\right)\right]\right]\;\mathrm{and}\;d\left(t\right)=d_{0}/\left[1-\sum_{i=1}^{n}k_{i}\left[1-\exp\left(-\frac{t}{\tau_{i}}\right)\right]\right],$$

wherein $c_{0}$ and $d_{0}$ denote the instantaneous moduli, i.e., the overall deviatoric and volumetric stiffnesses at infinite strain rate. In turn, the long-time moduli $c_{\infty }$ and $d_{\infty }$ for strain rate zero follow from

(11) $$c_{\infty }=c_{0}\left[1-\sum_{i=1}^{n}g_{i}\right]\;,\;d_{\infty }=d_{0}/\left[1-\sum_{i=1}^{n}k_{i}\right].$$

The material model sketched above is natively implemented in Abaqus and can be designed arbitrarily complex by choosing a large enough number of Maxwell elements. For simplicity, we assume all relaxation processes to be purely deviatoric, i.e., all parameters $k_{i}$ are set to zero in the following. Each optimization hence needs to determine the equilibrium modulus *E* which, together with ν, provides $c_{\infty }$ and $d_{\infty }$, as well as a pair of $\left(g_{i},\tau_{i}\right)$ for each Maxwell element. Optimization results for some increasingly complex models are presented below.

### 6.2. Results and Discussion

**Figure 22:**
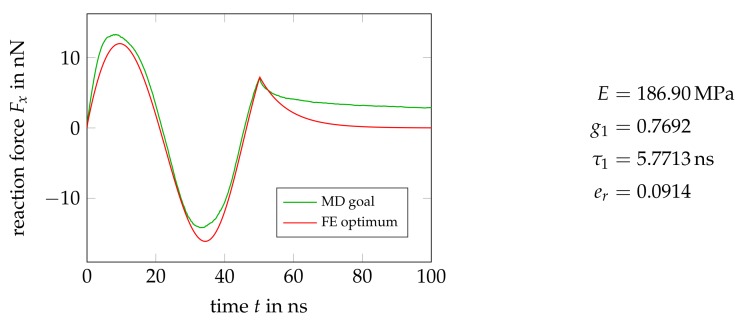
Optimal force history and material parameters from a calibration of the most simple viscoelastic model containing a single Maxwell element.

Figure 22 compares the force histories of the MD-simulation and an optimized FE-model containing a single Maxwell element. The resulting material parameters are reasonable and add up to an instantaneous stiffness of $c_{0}=E+c_{1}=809.8\;\mathrm{MPa}$, which is close to the values we have determined elsewhere [10,28,42]. All error values $e_{r}$ stated below to quantify the quality of the calibration are computed as the Euclidean norm of the difference between the MD goal and FE optimum vectors, divided by the number of data points in the goal, e.g., $e_{r}=\frac{1}{377}{∥F_{x}^{MD}-F_{x}^{FE}∥}_{{}_{2}}$. The loading-unloading cycle is reproduced acceptably, at least considering the low complexity of the material model used. The lack of plastic components in the model clearly becomes visible in the mismatch between the final relaxation curves of MD- and FE-simulation. From an applied strain of zero, a purely viscoelastic model will always relax to zero stress.

**Figure 23:**
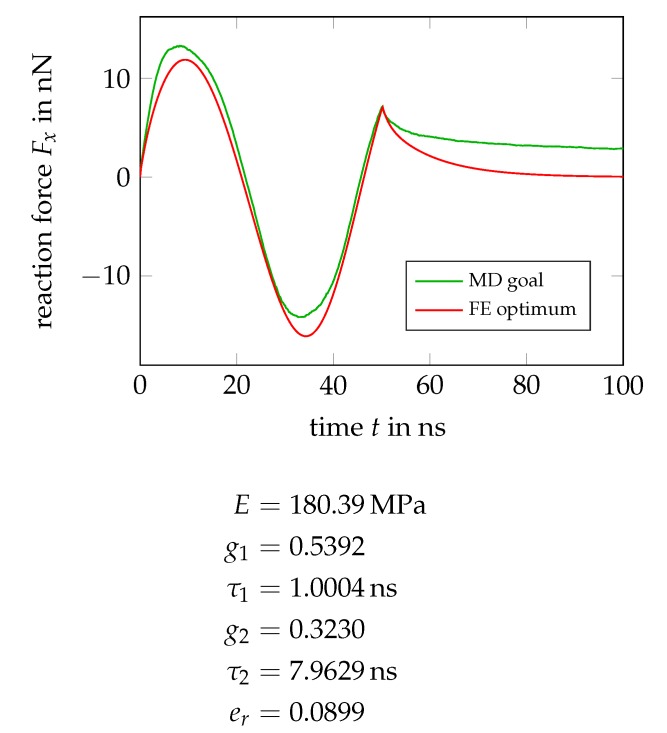
Optimal force history and material parameters from a calibration of a model containing a two Maxwell elements.

The addition of a second Maxwell element does not help in this regard, as shown in Figure 23. Merely the fit quality of the loading-unloading cycle slightly improves, compare the corresponding $e_{r}$ values, and one might conclude the presence of two relaxation mechanisms in the material with relaxation times differing by a factor of about eight.

**Figure 24:**
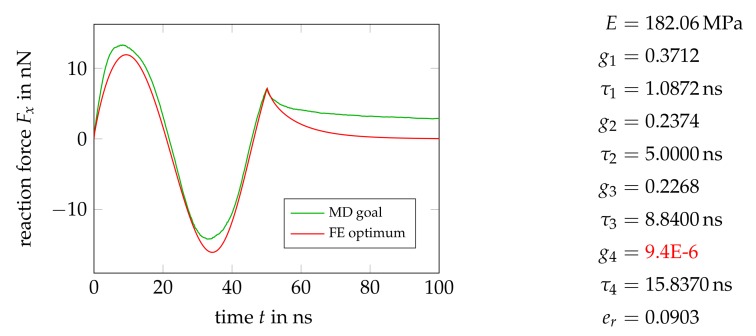
Optimal force history and material parameters from a calibration of a model containing a four Maxwell elements. Note that the fourth relaxator has almost been switched off by the optimizer.

Figure 24 contains the results for a model with four Maxwell elements, which obviously provides no further improvement. The fourth Maxwell element has even been switched off by the optimizer, as evident from the rather small $g_{4}$ value. That again confirms that two or three Maxwell elements are sufficient to capture the viscous characteristics of the material, additional model complexity is reasonable only for plastic behavior.

**Figure 25:**
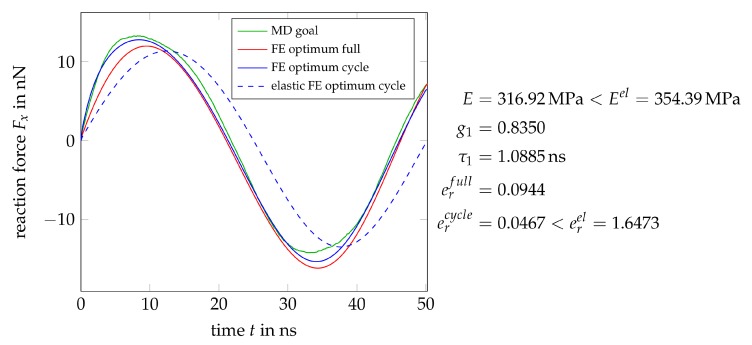
Optimal force history and material parameters for a model with one Maxwell element in comparison to a purely elastic model, calibrated with respect to only the loading-undloading cycle without the subsequent relaxation.

In applications where the long term behavior of the material is not of interest, a calibration of only the cyclic behavior would be sufficient. Therefore, this outlook is closed with a single Maxwell element model that is calibrated with respect to only the loading-unloading cycle, i.e., without consideration of the final relaxation phase as in all previous examples. The fit quality is twice as good as before, see Figure 25, in particular the first 30 ns are captured nearly perfectly. There is obviously some asymmetry between the tension and compression part that has to be accounted for by the choice of the plasticity formulation, which is anyhow required to capture the residual stresses after relaxation. Still, even in this simpler case, viscous behavior is required, compare the purely elastic optimization result in Figure 25. The preceding examples show that the MD deformation data presented in this work are very well suited to select and calibrate continuum mechanical material laws. The information contained in the MD data is obviously rather complex (asymmetric visco-elastoplastic behavior), so that the continuum mechanical modeling and calibration require some effort, which could at best be outlined here and will be the content of a subsequent contribution.

## 7. Conclusions and Outlook

In this contribution, we present a methodology to characterize the mechanical behavior of particle-based systems simulated with MD. It goes far beyond the investigation of rate and load dependencies of Young’s modulus and Poisson’s ratio, quantities that may from a continuum mechanical point of view characterize the mechanical behavior only for the linear regime. Here, however, we provide a sophisticated methodology to obtain information about elastic, plastic, and viscous contributions for large strains and multiple loading conditions similar to those typically carried out in experiments.

Based on our uniaxial tension tests using MD simulations, we can identify suitable parameters for the time discretization of our simulation set up. Furthermore, we prove the material to behave isotropic and investigate the influence of the applied strain rate and strain amplitude. For small deformations, we observe a predominantly elastic range. Time proportional tests, however, reveal a strain rate dependency which is typical for viscous materials. The stress-strain hystereses obtained in time periodic simulations indicate inelastic material behavior, which is caused by viscous as well as plastic effects as shown in relaxation and creep tests. As a conclusion, our results indicate that the present PS exhibits slight viscoelastic characteristics for small strains, whereas elastic viscoplasticity is observed for larger deformations. According to the classification scheme of Haupt [12], the observed strain rate dependence and quasi-static hystereses imply viscoplasticity and thus further supports our conclusion. Note that the methodology introduced and applied in this work is applicable to analyze the material behavior of any material that can be modeled with particle-based simulations.

In a next step, a continuum mechanical constitutive model has to be found as exemplarily shown in Section 6. In particular, this model has to precisely capture the mechanical behavior of the MD system of interest, including elasticity and inelasticity which requires a far more complex constitutive law. The subsequent identification of its material parameters will be facilitated by the large amount of data generated in this contribution. In the future we want to extend the MD code IBIsCO to allow for the examination of shear deformations in order to get a complete picture of the characteristics of the material under consideration. These findings will then be integrated into the already identified material laws, e.g., for PS, in order to further refine them.

As mentioned in the introduction, such material laws are particularly needed in partitioned-domain multiscale techniques, like the Capriccio method introduced by Pfaller et al. [9,28,42], but are also a necessity to correlate findings from MD simulations with those obtained from continuum mechanical computations and experimental evidence. Beyond this, developing and adjusting MD models focusing on their capability to reproduce mechanical material properties require a tool as presented here.

## Figures and Tables

**Table 1:** MD set up: time step Δt, temperature coupling time $t_{T}$, pressure coupling time $t_{p}$ and isothermal compressibility $\beta_{T}$.

Δt	5 $\times \;10^{-3}$ ps
$t_{T}$	0.2 ps
$t_{p}$	5 ps
$\beta_{T}$	1 $\times \;10^{-6}$ kPa

**Table 2:**
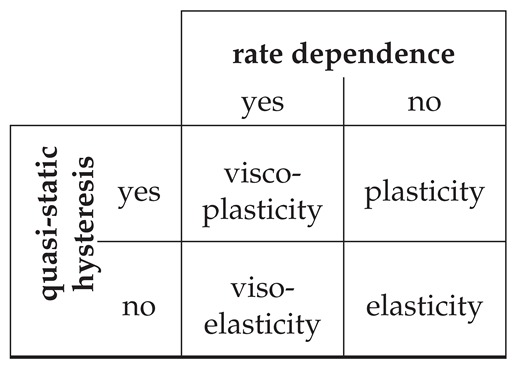
Classification scheme to characterize material behavior introduced by Haupt [12].

**Table 3:** Parameters for (a) time proportional and (b) time periodic loading: maximum stretch $\lambda_{i}^{\max}$, stretch amplitude $\lambda_{i}^{a}$, stretch rate ${\dot{\lambda }}_{i}$, maximum stretch rate ${{\dot{\lambda }}_{i}}^{\max}$, stretch increment per load step $▴\lambda_{i}$, number of timesteps per load step ▴n and time step Δt.

**(a) Time Proportional Load**	
$\lambda_{i}^{\max}$	1.08
${\dot{\lambda }}_{i}$	0.001 … 0.2 $ns^{-1}$
$▴\lambda_{i}$	$10^{-3}$
▴n	20000 , 40000 , 80000
Δt	$6.25\;\cdot \;10^{-2}$ … 50 fs
**(b) Time Periodic Load**	
$\lambda_{i}^{a}$	0.01 … 0.08
${\left|{\dot{\lambda }}_{i}\right|}^{\max}$	0.001 … 0.2 $ns^{-1}$
${\left|▴\lambda_{i}\right|}^{\max}$	$10^{-3}$
▴n	20000
Δt	$2.5\;\cdot \;10^{-1}$ … 50 fs

**Table 4:** Extrapolation parameters: (a) relaxation with $a=\sigma_{xx}\left(t\to \infty \right)$ and $bc^{-1}=\sigma_{xx}\left(t=0\right)$ and (b) creep with $a=E_{xx}\left(t\to \infty \right)$ and $bc^{-1}=E_{xx}\left(t=0\right)$.

**(a) Relaxation**			
$E_{xx}^{a}$	**a**	**b**	**c**
**%**	**–**	**ns**	**ns**
2	0.142	0.60	0.607
4	1.435	23.75	5.474
6	3.494	62.91	6.114
8	5.998	104.9	6.819
**(b) Creep**			
$E_{xx}^{a}$	**a**	**b**	**c**
**%**	**MPa ns** ${}^{-2}$	**MPa ns** ${}^{-1}$	**ns**
2	0.0247	0.350	2.4
4	0.1721	2.943	4.908
6	0.8959	1.359	7.459
8	2.3630	2.621	6.738

## Abbreviations

The following abbreviations are used in this manuscript:

CG	Coarse-Grained
MD	Molecular Dynamics
PS	Polystyrene
